# Supplementary material for: Distinct roles of cortical layer 5 subtypes in associative learning
Source: Nat Commun. 2026 Mar 20;17:2648. doi: 10.1038/s41467-026-68307-5 (PMC13004866; doi:10.1038/s41467-026-68307-5)
Supplement: Supplementary file 1 — Supplementary Information [file 41467_2026_68307_MOESM1_ESM.pdf]

Supplementary Information for:

Distinct roles of cortical layer 5 subtypes in associative learning

Sara Moberg[1,2], Michele Garibbo[3,4]*, Camille Mazo[5]*, Ariel Gilad[6], Dietmar Schmitz[1,7,8,9,10,11], Rui Ponte Costa[3], Matthew E. Larkum[2]†, and Naoya Takahashi[5]†

[1]Charité - Universitätsmedizin Berlin, corporate member of Freie Universität Berlin and Humboldt Universität zu Berlin, Einstein Center for Neurosciences Berlin, 10117 Berlin, Germany
[2]Institute for Biology, Humboldt University of Berlin, 10117 Berlin, Germany
[3]Centre for Neural Circuits and Behaviour, Department of Physiology, Anatomy and Genetics, University of Oxford, Oxford, United Kingdom
[4]Bristol Computational Neuroscience Unit, Intelligent Systems Lab, Faculty of Engineering, University of Bristol, Bristol, BS8 1TH, United Kingdom
[5]University of Bordeaux, CNRS, Interdisciplinary Institute for Neuroscience, IINS, UMR 5297, 33000 Bordeaux, France
[6]Department of Medical Neurobiology, Faculty of Medicine, Institute for Medical Research Israel-Canada (IMRIC), The Hebrew University of Jerusalem, Jerusalem, Israel
[7]Charité - Universitätsmedizin Berlin, corporate member of Freie Universität Berlin and Humboldt Universität zu Berlin, Neuroscience Research Center, 10117 Berlin, Germany
[8]Charité - Universitätsmedizin Berlin, corporate member of Freie Universität Berlin and Humboldt Universität zu Berlin, NeuroCure Cluster of Excellence, 10117 Berlin, Germany.
[9]Bernstein Center for Computational Neuroscience Berlin, Berlin 10115, Germany
[10]German Center for Neurodegenerative Diseases (DZNE) Berlin, Berlin 10117, Germany
[11]Max-Delbrück Center for Molecular Medicine in the Helmholtz Association, Berlin 13125, Germany

*These authors contributed equally to this work.
†Correspondence: Naoya Takahashi (naoya.takahashi@u-bordeaux.fr) and Matthew E. Larkum (matthew.larkum@hu-berlin.de)

This PDF file includes:
Supplementary Figures 1–6

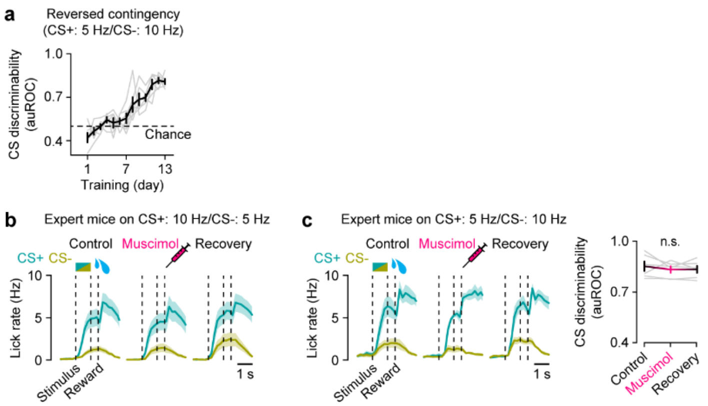

Supplementary Fig. 1 | S1 is required for learning the reversed contingency but not for performing the task once learned.

(**a**) Evolution of behavioral discrimination between conditioned stimuli with the reversed CS contingency (CS+: 5 Hz; CS−: 10 Hz) over 13 days of training ($n = 6$ mice; $P = 7.1 \times 10^{-17}$, $F = 20.45$; one-way repeated-measure ANOVA). Gray lines, individual mice.

(**b**) Effect of S1 inactivation on lick rates for CS+ (blue) and CS− (yellow) trials in expert mice trained on the original contingency ($n = 9$ mice).

(**c**) Left, same as **b** but in expert mice trained on the reversed contingency ($n = 6$ mice). Right, behavioral performance of the same mice injected with muscimol ($n = 6$ mice; $P = 0.50$, $F = 0.75$; one-way repeated-measure ANOVA). Gray lines, individual mice.

Data are presented as mean ± SEM. Source data are provided as a Source Data file.

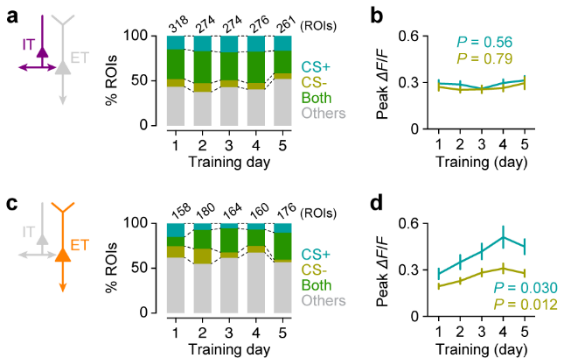

Supplementary Fig. 2 | Learning-related changes in IT and ET neuron responses to conditioned stimuli.

(**a**) Fractions of IT neurons responding to CS+ only (blue), CS− only (yellow), or both stimuli (green) for Day 1-5 ($P = 0.14$, $\chi^2 = 17.24$; 4×5 χ^2 test).

(**b**) Peak responses to CS+ (blue; $F = 0.75$; one-way ANOVA) and CS− (yellow; $F = 0.43$; one-way ANOVA) across training days.

(**c–d**) Same as **a** and **b** but for ET neurons. (**c**) $P = 1.8 \times 10^{-7}$, $\chi^2 = 55.02$. (**d**) CS+: $F = 2.72$; CS−: $F = 3.29$. Data are presented as mean ± SEM. Source data are provided as a Source Data file.

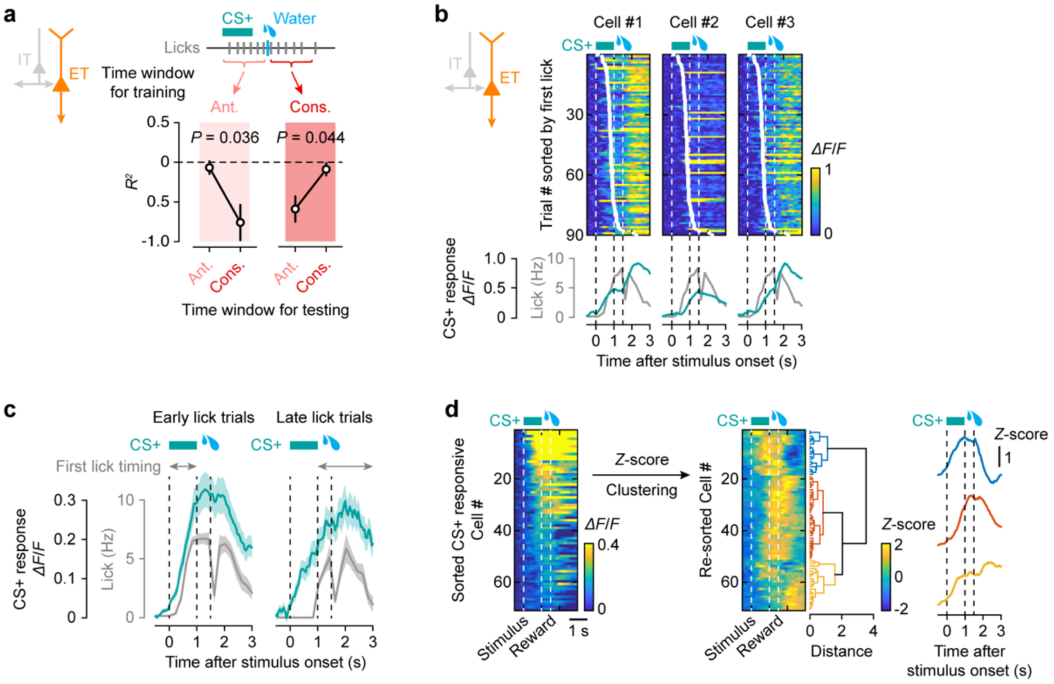

Supplementary Fig. 3 | ET neuronal signals are not simply attributable to lick-related motor activity.
(**a**) SVM regression performance of ET neuronal activity in decoding lick rates in the anticipatory (Ant.) lick time window and consummatory (Cons.) lick time window in CS+ trials ($n = 6$ mice). Left, the decoders were trained on ET neuronal responses in the anticipatory lick time window and tested on the same time window vs. the consummatory lick time window. Right, the decoders were trained on ET neuronal responses in the consummatory lick time window and tested on the same time window vs. the anticipatory lick time window.
(**b**) Top, calcium responses of three example ET neurons in CS+ trials, sorted based on the lick onset. Bottom, averaged ET neuronal responses (blue) and lick activity (gray).
(**c**) Left, calcium responses in CS+ trials where mice initiated licking during the stimulus presentation time window, averaged across ET neurons responding to CS+ ($n = 71$ neurons from 6 mice; 75–92 early lick trials per mouse). Right, calcium responses in CS+ trials where mice licked after the stimulus presentation time window ($n = 71$ neurons from 6 mice; 8–25 late lick trials per mouse). Note that there was calcium activity in the stimulus window despite the lack of licking. Blue trace, ET neuronal calcium responses; gray trace, average lick rates.
(**d**) Left, Heatmap of calcium responses from ET neurons responsive to CS+ ($n = 71$ neurons from 6 mice) during CS+ trials on Day 5, sorted by their mean activity within the anticipatory licking window (before reward delivery). Right, heatmap of *z*-scored responses of the same neurons, sorted by hierarchical clustering. Three clusters are highlighted, and their corresponding mean traces are shown next to the dendrogram. Notably, neurons in the first cluster (blue) were preferentially active during the anticipatory licking window but not during the consummatory licking window (after reward delivery).
Data are presented as mean ± SEM.

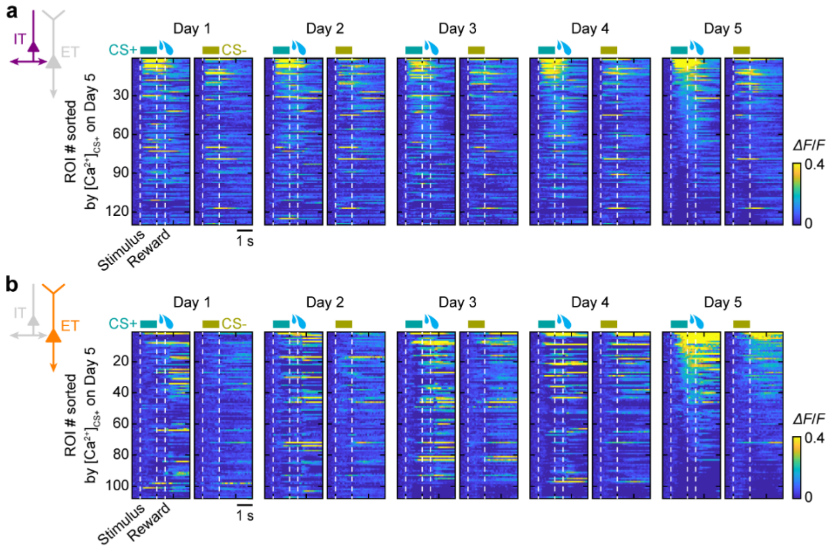

Supplementary Fig. 4 | Responses of tracked IT and ET neurons to conditioned stimuli during learning.

(**a**) Heatmaps of CS responses of tracked IT neurons sorted by their mean activity in CS+ trials on Day 5 (1.5 s from stimulus onset, n = 130 tracked IT neurons).

(**b**) Same as in **a** but for tracked ET neurons (n = 108 tracked ET neurons).

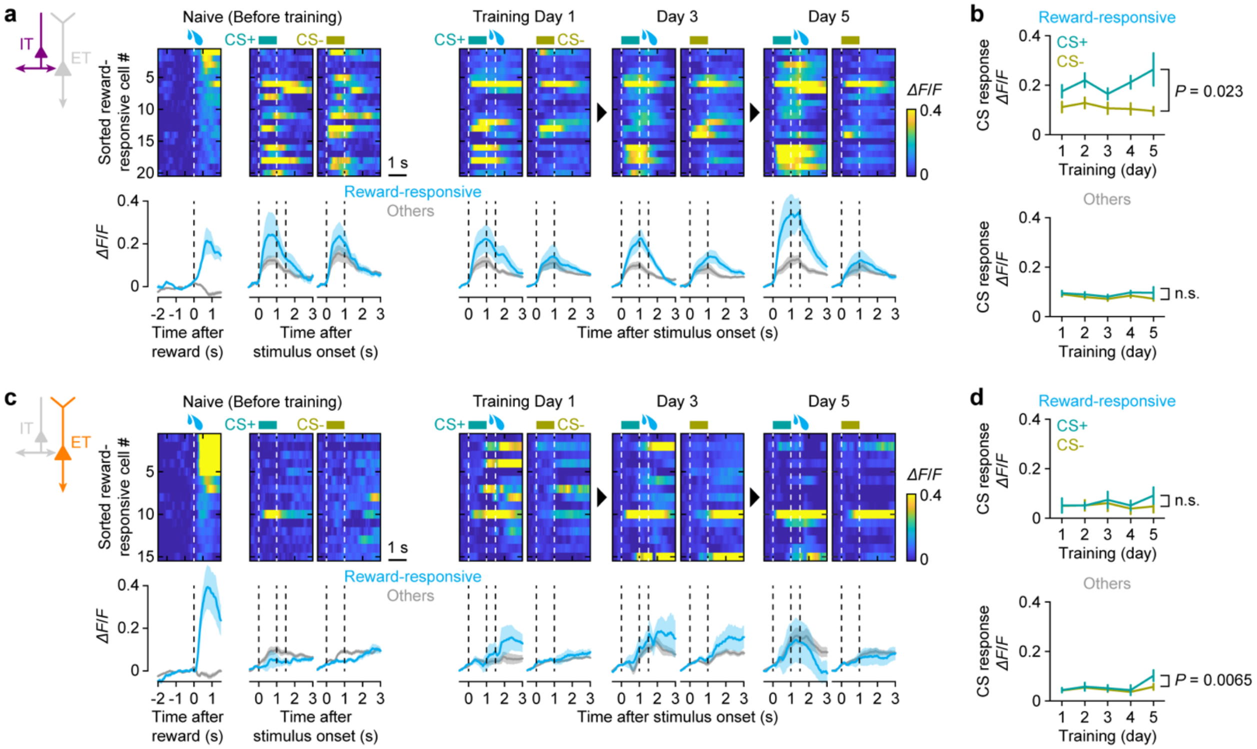

Supplementary Fig. 5 | Responses of reward-responding IT and ET neurons to conditioned stimuli before and during learning.

(**a**) Top, heatmaps of responses of tracked IT neurons that responded to reward before learning (n = 20 neurons from 5 mice), sorted by their mean activity: their responses to water reward and stimuli before learning and those in CS+ and CS− trials during learning. Bottom, average responses of tracked reward-responsive IT neurons (blue) and other IT neurons (n = 83 neurons; gray).

(**b**) Top, average responses of tracked reward-responsive IT neurons to CS+ (blue) and CS− (yellow) across training (F = 3.02; two-way repeated-measure ANOVA). Bottom, average CS responses of non-reward-responsive (other) IT neurons across training (P = 0.68, F = 0.58; two-way repeated-measure ANOVA).

(**c**, **d**) Same as **a** but for tracked ET neurons (n = 15 reward-responsive neurons and 76 other neurons from 6 mice). (**d**) Top, P = 0.37, F = 1.09. Bottom, F = 3.64.

Data are presented as mean ± SEM. Source data are provided as a Source Data file.

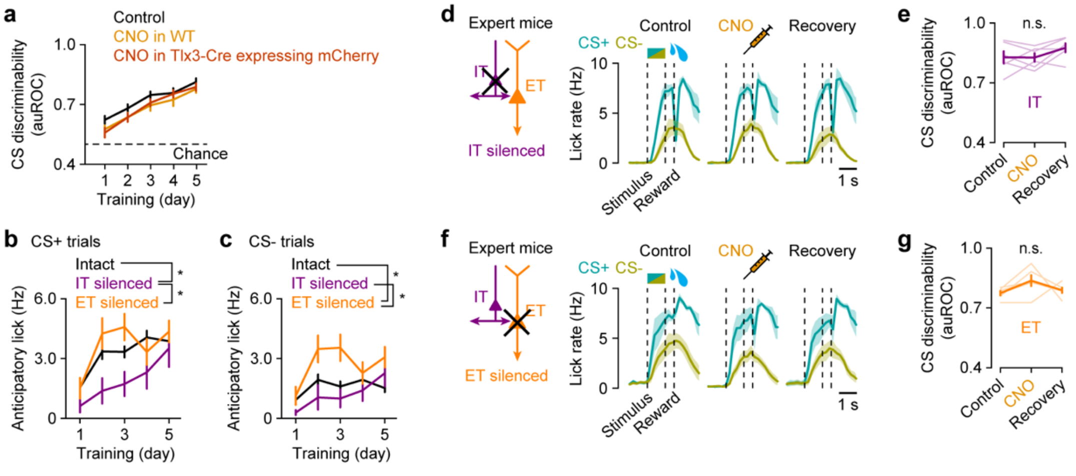

Supplementary Fig. 6 | Effect of IT and ET neuronal inactivation on behavior during and after learning.

(**a**) Behavioral performance of control mice with intact S1 ($n = 20$ mice), wild-type mice injected with CNO ($n = 6$ mice) and mCherry-expressing Tlx3-Cre mice injected with CNO ($n = 6$ mice; $P = 0.17$, $F = 1.86$; two-way mixed ANOVA).

(**b**) Anticipatory lick rates for CS+ trials for mice with intact S1 ($n = 20$ mice), mice with silenced IT neurons, ($n = 6$ mice) and mice with silenced ET neurons ($n = 6$ mice; $P = 0.031$, $F = 3.92$; two-way mixed ANOVA with post hoc Tukey-Kramer test). $^{*}P < 0.05$.

(**c**) Anticipatory lick rates for CS− trials for mice with intact S1 ($n = 20$ mice), mice with silenced IT neurons, ($n = 6$ mice) and mice with silenced ET neurons ($n = 6$ mice; $P = 0.017$, $F = 4.71$; two-way mixed ANOVA with post hoc Tukey-Kramer test). $^{*}P < 0.05$.

(**d**) Lick rates for CS+ (blue) and CS− (yellow) trials in expert mice with silenced IT neurons for Days 1–5 ($n = 6$ mice). Gray lines, individual mice.

(**e**) Behavioral performance of expert mice with silenced IT neurons ($n = 6$ mice; $P = 0.077$, $F = 3.36$; one-way repeated-measure ANOVA)

(**f–g**) Same as **d** and **e** but in expert mice with silenced ET neurons. (**g**) $n = 6$ mice; $P = 0.13$, $F = 2.56$.

Data are presented as mean ± SEM. Source data are provided as a Source Data file.
