# Supplementary material for: Distinct roles of cortical layer 5 subtypes in associative learning
Source: Nat Commun. 2026 Mar 20;17:2648. doi: 10.1038/s41467-026-68307-5 (PMC13004866; doi:10.1038/s41467-026-68307-5)
Supplement: Supplementary file 2 — Reporting Summary [file 41467_2026_68307_MOESM2_ESM.pdf]

nature portfolio

Corresponding author(s): Naoya Takahashi
Last updated by author(s): Dec 4, 2025

Reporting Summary

Nature Portfolio wishes to improve the reproducibility of the work that we publish. This form provides structure for consistency and transparency in reporting. For further information on Nature Portfolio policies, see our Editorial Policies and the Editorial Policy Checklist.

Statistics

For all statistical analyses, confirm that the following items are present in the figure legend, table legend, main text, or Methods section.

n/a	Confirmed	
☐	☒	The exact sample size (*n*) for each experimental group/condition, given as a discrete number and unit of measurement
☐	☒	A statement on whether measurements were taken from distinct samples or whether the same sample was measured repeatedly
☐	☒	The statistical test(s) used AND whether they are one- or two-sided<br>*Only common tests should be described solely by name; describe more complex techniques in the Methods section.*
☐	☒	A description of all covariates tested
☐	☒	A description of any assumptions or corrections, such as tests of normality and adjustment for multiple comparisons
☐	☒	A full description of the statistical parameters including central tendency (e.g. means) or other basic estimates (e.g. regression coefficient) AND variation (e.g. standard deviation) or associated estimates of uncertainty (e.g. confidence intervals)
☐	☒	For null hypothesis testing, the test statistic (e.g. *F*, *t*, *r*) with confidence intervals, effect sizes, degrees of freedom and *P* value noted<br>*Give P values as exact values whenever suitable.*
☒	☐	For Bayesian analysis, information on the choice of priors and Markov chain Monte Carlo settings
☒	☐	For hierarchical and complex designs, identification of the appropriate level for tests and full reporting of outcomes
☐	☒	Estimates of effect sizes (e.g. Cohen's *d*, Pearson's *r*), indicating how they were calculated

Our web collection on statistics for biologists contains articles on many of the points above.

Software and code

Policy information about availability of computer code

Data collection	ScanImage (Vidrio Technologies; https://vidriotechnologies.com/)
Data analysis	Matlab2021b (data and statistical analysis)<br>Suite2p (https://github.com/MouseLand/suite2p)

For manuscripts utilizing custom algorithms or software that are central to the research but not yet described in published literature, software must be made available to editors and reviewers. We strongly encourage code deposition in a community repository (e.g. GitHub). See the Nature Portfolio guidelines for submitting code & software for further information.

Data

Policy information about availability of data

All manuscripts must include a data availability statement. This statement should provide the following information, where applicable:

- Accession codes, unique identifiers, or web links for publicly available datasets
- A description of any restrictions on data availability
- For clinical datasets or third party data, please ensure that the statement adheres to our policy

The data used in this study are available via Zenodo at https://doi.org/ 10.5281/zenodo.17720603. Source data are provided with this paper.

Research involving human participants, their data, or biological material

Policy information about studies with human participants or human data. See also policy information about sex, gender (identity/presentation), and sexual orientation and race, ethnicity and racism.

Reporting on sex and gender	N/A
Reporting on race, ethnicity, or other socially relevant groupings	N/A
Population characteristics	N/A
Recruitment	N/A
Ethics oversight	N/A

Note that full information on the approval of the study protocol must also be provided in the manuscript.

Field-specific reporting

Please select the one below that is the best fit for your research. If you are not sure, read the appropriate sections before making your selection.

☒ Life sciences ☐ Behavioural & social sciences ☐ Ecological, evolutionary & environmental sciences

For a reference copy of the document with all sections, see nature.com/documents/nr-reporting-summary-flat.pdf

Life sciences study design

All studies must disclose on these points even when the disclosure is negative.

Sample size	Sample size was estimated based on the expected effect size based on similar studies (Takahashi et al., 2020; Benezra et al 2024) and the current standard in mouse neuroscience studies .
Data exclusions	Poor data quality usually originates from movement artifacts during neuronal imaging of awake mice performing a behavior. Two-photon recordings with poor signal/data quality were excluded as assessed by visual inspection of the registered time-series images and registration metrics. Data exclusion was done prior to analysis, in order not to bias exclusion criteria.
Replication	Experiments were performed independently in different mice and statistics were performed across mice in most cases.
Randomization	Stimuli presentations were randomized and mice were randomly selected for the different experimental groups.
Blinding	Although the experimenter can tell during recording whether IT or ET neurons were recorded, all recorded neurons were analyzed in the same way and the same statistical tests were performed in both groups. The same applies for the behavioral analysis of all the animal groups. Thus, the measurements are not affected by the lack of blinding.

Reporting for specific materials, systems and methods

We require information from authors about some types of materials, experimental systems and methods used in many studies. Here, indicate whether each material, system or method listed is relevant to your study. If you are not sure if a list item applies to your research, read the appropriate section before selecting a response.

Materials & experimental systems

n/a	Involved in the study	
☒	☐	Antibodies
☒	☐	Eukaryotic cell lines
☒	☐	Palaeontology and archaeology
☐	☒	Animals and other organisms
☒	☐	Clinical data
☒	☐	Dual use research of concern
☒	☐	Plants

Methods

n/a	Involved in the study	
☒	☐	ChIP-seq
☒	☐	Flow cytometry
☒	☐	MRI-based neuroimaging

Animals and other research organisms

Policy information about studies involving animals; ARRIVE guidelines recommended for reporting animal research, and Sex and Gender in Research

Laboratory animals	Animals used in this study were of the species Mus musculus. The strains used were as followed: C57BL/6J, Sim1-Cre (KJ18) (MMRRC, no. 031742-UCD), Tlx3-Cre (PL56) (MMRRC, no. 041158-UCD). Age: P60 - P80
Wild animals	No wild animals were used in this study.
Reporting on sex	Data was collected from 53 males and 24 females. All of the mice groups were bred in-house and experiments were performed in both males or females, depending on availability.<br>A detailed division of gender of the mice per experiments are as followed:<br>- Behavioral test in mice with intact S1: 16 (male), 4 (female)<br>- Muscimol injection during learning: 6, 0<br>- Muscimol injection in expert mice: 6, 3<br>- CNO injection in Tlx3-Cre mice expressing hM4Di: 3, 3<br>-CNO injection in Sim1-Cre mice expressing hM4Di: 6, 0<br>- Calcium imaging in Tlx3-Cre mice: 4, 1<br>- Calcium imaging in Sim1-Cre mice: 5, 1<br>- Ringer injection during learning: 6, 0<br>- CNO injection in wild-type mice expressing hM4Di: 6, 0<br>- CNO injection in Tlx3-Cre mice expressing mCherry: 1, 5<br>- Behavioral test in mice trained on the reversed contingency: 3, 3<br>- Muscimol injection in expert mice trained on the reversed contingency: 0, 6
Field-collected samples	This study did not use field-collected samples.
Ethics oversight	All experiments were conducted following the guideline given by Landesamt für Gesundheit und Soziales Berlin (LAGeSo) and were approved by this authority (protocol number: G0278/16 & G0161/21).

Note that full information on the approval of the study protocol must also be provided in the manuscript.

Plants

Seed stocks	*Report on the source of all seed stocks or other plant material used. If applicable, state the seed stock centre and catalogue number. If plant specimens were collected from the field, describe the collection location, date and sampling procedures.*
Novel plant genotypes	*Describe the methods by which all novel plant genotypes were produced. This includes those generated by transgenic approaches, gene editing, chemical/radiation-based mutagenesis and hybridization. For transgenic lines, describe the transformation method, the number of independent lines analyzed and the generation upon which experiments were performed. For gene-edited lines, describe the editor used, the endogenous sequence targeted for editing, the targeting guide RNA sequence (if applicable) and how the editor was applied.*
Authentication	*Describe any authentication procedures for each seed stock used or novel genotype generated. Describe any experiments used to assess the effect of a mutation and, where applicable, how potential secondary effects (e.g. second site T-DNA insertions, mosiacism, off-target gene editing) were examined.*
